# A Three-Step Reconstruction of the Breast in a Patient With Congenital Unilateral Amastia

**DOI:** 10.7759/cureus.18540

**Published:** 2021-10-06

**Authors:** Aysuna Galandarova, Vagif Kalender, Arturan Ibrahimli, Gunel Guliyeva

**Affiliations:** 1 Plastic and Reconstructive Surgery, Ankara Yildirim Beyazit University, Ankara, TUR; 2 Plastic and Reconstructive Surgery, Azerbaijan Medical University, Baku, AZE; 3 Medicine, Ankara University Faculty of Medicine, Ankara, TUR

**Keywords:** breast reconstruction, nipple–areola complex reconstruction, areola reconstruction, nipple reconstruction, absence of nipple-areola, congenital amastia

## Abstract

Amastia refers to a condition where breast tissue, nipples, and areoles are congenitally absent, and it can affect one (unilateral) or both (bilateral) breasts. Congenital amastia is a rare condition with only 34 reported cases in the literature. In this case, we report a 17-year-old female with congenital unilateral amastia of the right breast. She came to our clinic due to a cosmetic view of this defect, which was bothering her, and greatly reducing the overall quality of her life. Our patient's physical examination revealed the absence of right breast, and there was no other obvious physical or anatomical abnormality. The defect was successfully reconstructed in three steps. Firstly, 200 cc adipose tissue was transferred under the skin before inserting the breast implant due to increasing the thickness between the skin and the nipple-areola. Secondly, after four months breast implant was inserted. Finally, the patient's right nipple-areola complex (NAC) was reconstructed with a skate flap.

## Introduction

Amastia refers to a condition where breast tissues, nipples, and areolas are absent. It can be either unilateral or bilateral and can be isolated or part of several syndromes [1]. Poland sequence (PS) is the most typical cause of unilateral amastia. It is characterized by unilateral absence or hypoplasia of the breast and the pectoralis muscle, anterior rib abnormalities, syndactyly, and brachydactyly [2]. The incidence of PS is between 1/30,000 and 1/32,000 live births [3]. Deformity or absence of the breast significantly impairs the quality of life of the patients. Therefore, early detection and management of breast anomalies are crucial for the patients.

## Case presentation

**Figure 1:**
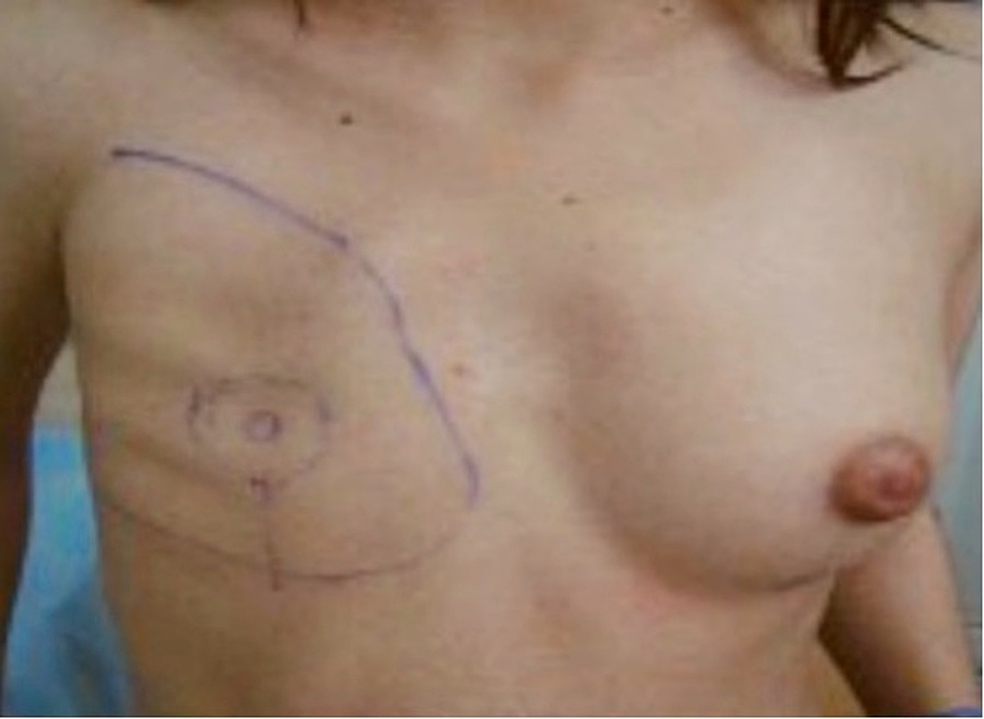
Patient before reconstruction.

**Figure 2:**
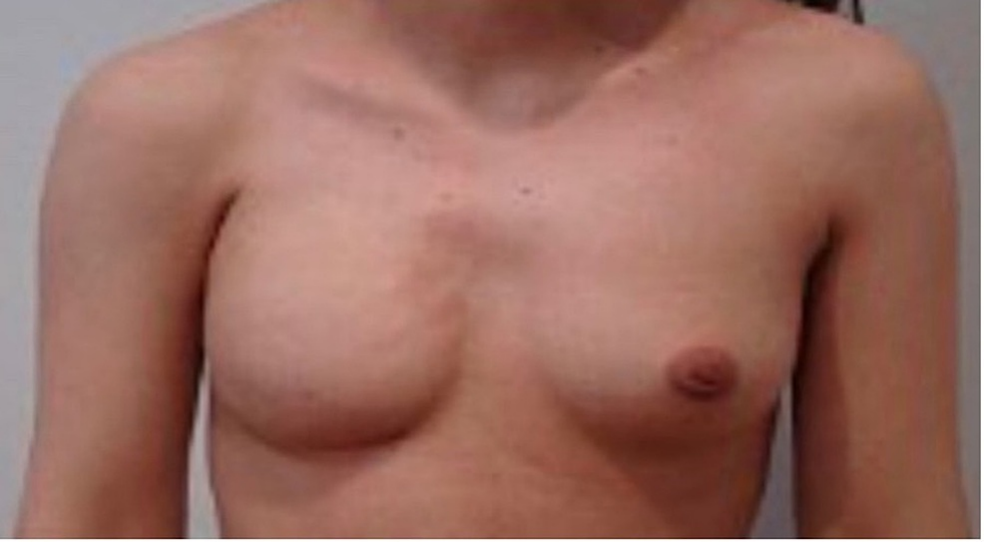
Patient after placement of implant.

**Figure 3:**
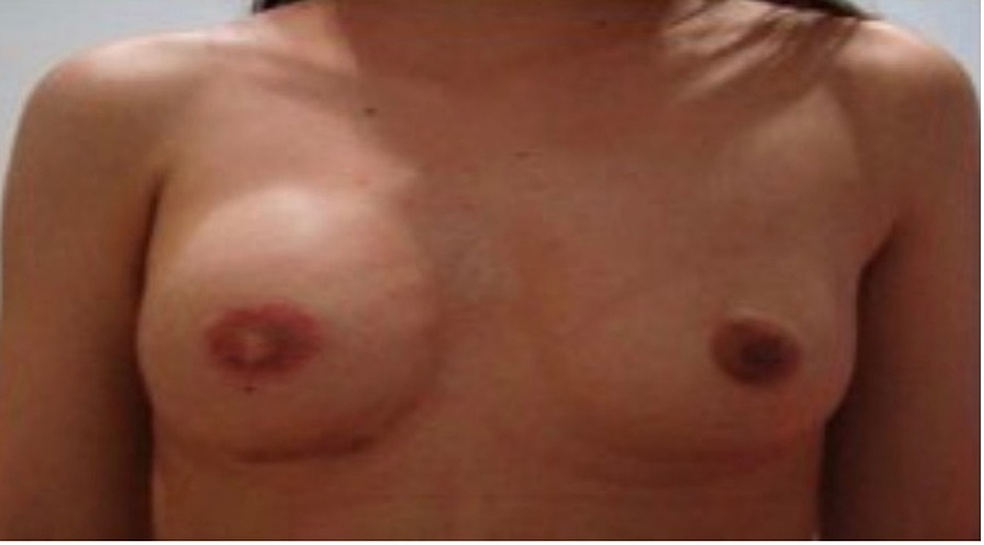
Patient after final NAC reconstruction. NAC, nipple-areola complex

A 17-year-old female patient presented with unilateral congenital absence of right breast tissue, areola, and nipple. This deformity adversely affected the patient's daily life, and she did not even want to go to school. Therefore, she requested consultation regarding the deformity of her right breast. She has no significant family history and medical condition. The physical examination revealed intact pectoralis major muscle and no other deformity (Figure 1). In stage 1, we took the 200 cc adipose tissue from the skin of the abdominal region on which side there was no mammary gland and transferred it under the skin in that area by making incisions in inconspicuous areas to minimize visible scars. The purpose was to thicken the skin on the right side before placing the breast implant to reduce the risk of implant extrusion. This operation was performed under local anesthesia. Approximately four months after the first stage operation, under general anesthesia, an incision was made to the pectoralis major muscle with a 4 cm incision by entering through the inframammary fold. After the incision was made, considering that the left breast of the adolescent girl will grow in the future, a slightly larger breast implant (320 cc) was inserted into a pocket under the pectoral muscle (Figure 2). The patient was called to our clinic again after four months to reconstruct her right nipple-areola complex (NAC), which is the final stage of breast reconstruction. The NAC was reconstructed with a skate flap with a full-thickness graft taken from the skin of the labia majors under local anesthesia (Figure 3).

## Discussion

The absence of breast tissue is the least frequent of all congenital breast anomalies. Trier conducted a thorough study of the literature and discovered 43 cases for which data were available. Bilateral absence of breasts with congenital ectodermal abnormalities (seven cases), unilateral absence of breasts (20 cases), and bilateral absence of breasts (16 cases) with various related abnormalities were all reported [4]. Congenital amazia is defined as the absence of glandular breast tissue with a normal NAC. Related conditions include congenital athelia where only the NAC is absent, amastia where both the mammary tissue and the NAC are absent, and breast hypoplasia where there is a deficiency of mammary tissue [5]. Congenital ectodermal defects are also associated with amastia and affect both male and female patients equally. Bilateral amastia with accompanying skin abnormalities, skin appendages (hair, eccrine glands, and sebaceous glands), teeth, and nails is a common patient presentation [5-6]. No distinctive body or anatomic abnormalities were found in our patient, as mentioned earlier. In relatively few cases, after breast implant reconstruction surgery, the implant's surface or tissue expander penetrates the skin and becomes exposed. However, implant extrusion can occur if the skin over the implant is too thin and there is little coverage left for the implant, or if the implant used to reconstruct the breast is larger and puts too much pressure on the skin [7]. In this case, the reason for performing this surgery in three steps was to first increase the thickness between the skin and the nipple-areola by transferring adipose tissue under the skin and then place the implant to reduce the risk of the implant extrusion and increase NAC fullness. Our approach gave us confidence that our patient will not have any cosmetic or mental difficulties connected to her breast deformity for many years. In the future, the conduction of case-control studies regarding the advantages and disadvantages of three-step reconstructive breast implant surgeries and traditional surgeries in patients with amastia would give us a broad approach to reconstructive breast implant surgeries.

## Conclusions

To conclude, transferring the adipose tissue before placing the implant gave us confidence that we minimized the risk of implant extrusion by increasing the thickness between the skin and the nipple. Moreover, a successful cosmetic result was achieved by this surgical procedure. By reconstructing the 17-year-old patient's right breast, her self-image, confidence, and overall feelings were boosted. In addition, our patient was relieved mentally, and her issues about her body have vanished after the operations.
